# Exploring the relationship between mental health and dialect use among Chinese older adults: a moderated mediation estimation

**DOI:** 10.3389/fpsyg.2023.1177984

**Published:** 2023-07-28

**Authors:** Tianxin Li, Jin Li, Xigang Ke

**Affiliations:** ^1^Department of Literature, Shaanxi Normal University, Xi'an, China; ^2^International School of Chinese Studies, Shaanxi Normal University, Xi'an, China

**Keywords:** dialects, income inequality, subjective well-being, mental health, moderated mediation

## Abstract

**Background:**

Mental health, conceptualized as psychological status that includes rational cognition, emotional stability, and interpersonal harmony, is highly relevant to the expected health and well-being of all humans. China is facing the dual risk of increased aging and mental health disorders in older adults, while the established studies have rarely focused on the influence of dialect on the mental health of Chinese older adults. The present study aims to capture the relationship between dialect and mental health in Chinese older adults.

**Methods:**

We use cross-sectional data from the nationally representative China Family Panel Studies, which encompasses the dialect use, mental health, and other socioeconomic features of 4,420 respondents. We construct a moderated mediation model that uses dialects and mental health as the independent and dependent variables and income inequality and subjective well-being as the mediator and moderator to reveal the relationship between dialect and mental health in Chinese older adults.

**Results:**

(1) Dialects are shown to have a negative influence on the mental health of older adults in the current study (coefficient = −0.354, 95% CI = [−0.608, −0.097]). (2) Income inequality positively mediates the correlation between dialects and mental health (coefficient = 0.019, 95% CI = [0.010, 0.045]). (3) Subjective well-being negatively moderates the potential mechanism between dialects and mental health (coefficient = −0.126, 95% CI = [−0.284, −0.010]).

**Conclusion:**

The use of dialects is associated with worse mental health outcomes in Chinese older adults, while this negative influence is positively mediated by income inequality and negatively moderated by subjective well-being, simultaneously. This study contributes to the knowledge enrichment of government workers, older adults with mental disorders, medical staff, and other stakeholders.

## 1. Introduction

The world is witnessing an aging period, whereby the estimated number of older adults reached 1.07 billion as of 2020 and is predicted to exceed 1.82 billion by 2030 (Rudnicka et al., 2020). Accordingly, comprehensive health care for older adults is given high priority. Mental health (MH) for older adults in China is particularly important, given the increasing prevalence of aging and the large number of older adults suffering from mental illness, including depression, anxiety, and mood disorders (Brisset et al., 2014). MH disorders of older adults are more prevalent in developing or underdeveloped countries and regions, sharing higher morbidity and mortality, and posing multilevel and multifactorial social burden (Gureje, 2020). For example, the prevalence of depression in older adults is 7.8–34.8% in Asia, which is three times higher than in Europe (Mohd et al., 2019). The economic burden of MH disorders in Chinese older adults also deserves special attention. The MH disorder detection rate in China was reported as 33.0% in 2022 (Dadi et al., 2020; Cui et al., 2022); meanwhile, the annual cost of MH medical treatment is $3,665 per patient (Xu et al., 2016), which means the total estimated cost could exceed 1.46 trillion dollars. Therefore, clarifying the determinants of MH disorders for Chinese older adults is given high academic priority.

**Figure 1:**
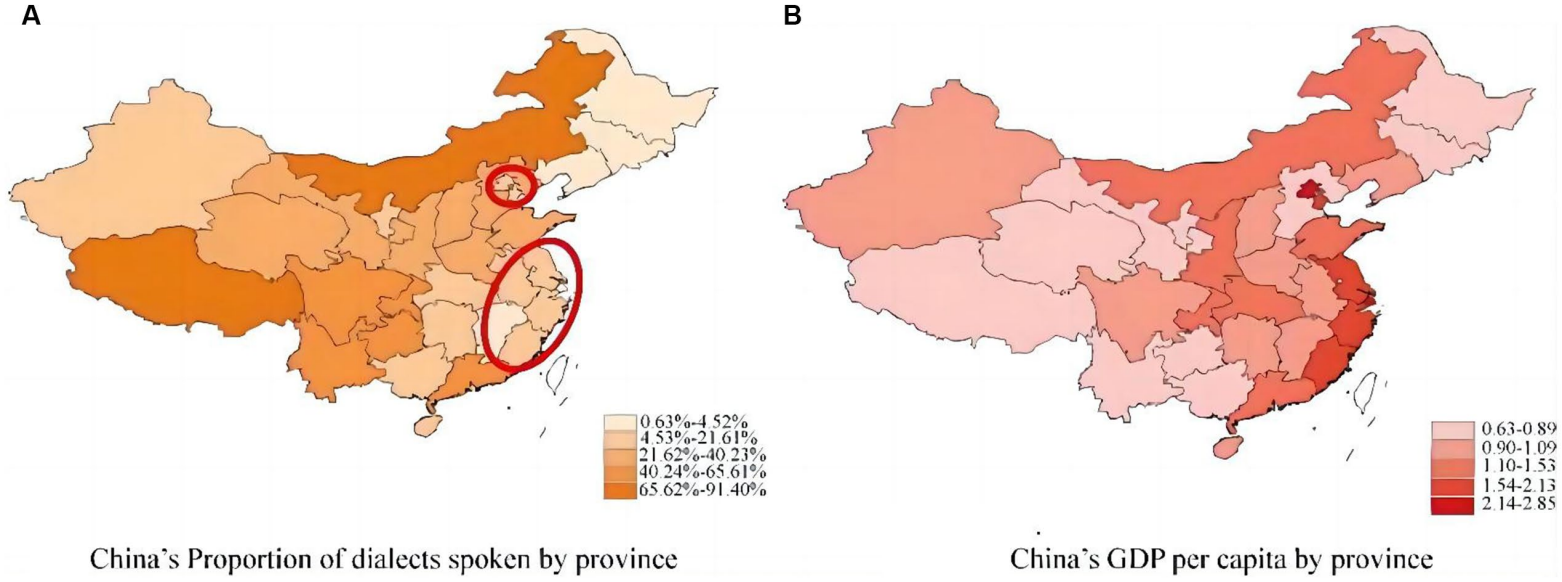
The proportion of dialect speakers and GDP *per capita*^abc^. ^a^The data is available at http://www.stats.gov.cn/sj/ndsj/2022/indexch.htm. ^b^The larger the value, the darker the color. ^c^ Beijing-Tianjin-Hebei region (political center of China, small red circle) and Yangtze River Delta region (economic center of China, large red circle): In these two regions, the proportion of dialect speakers was low, but the GDP per capita was high.

Previous studies have focused more on the protection of the physical health of older adults and have found that older adults are suffering from a variety of physical diseases such as diabetes, hypertension, cancer, and obesity, leading to their premature death and increasing all-cause mortality worldwide (Salihu et al., 2009; Uchmanowicz et al., 2019). However, the determinants of modifiable MH, especially the potential mechanism between determinants and MH is still vague. Several sporadic research studies have captured traditional MH disorder determinants like genes, smoking, diet, exercise, and pollutant exposure (Zaman et al., 2019; Firth et al., 2020). However, in China, one particular variable deserves special attention, namely the use of dialects. Dialects are conceptualized as the regional variants of a language that differ from a standard language (such as Mandarin) and are spoken only in specific regions (Francis, 2016). The language barriers that may come with dialect use are particularly salient in China due to the compounded risks posed by social inequality (Trudgill, 2012; Ohtani et al., 2015). From an academic perspective, dialect may shape MH disorders in older adults via the following mechanism: dialect is a signal that distinguishes between the socioeconomic statuses of individuals, and for older adults who are unable to integrate into normal social life due to language barriers, their MH might face potential risks (Smits and Gündüz-Hoşgör, 2003; Mueller Gathercole et al., 2010). Dialect use, together with an individual’s socioeconomic status, may limit an individual’s ability to participate in business or political activities, ultimately affecting their MH (Carlson and McHenry, 2006). We geographically depict the distribution of dialects and socioeconomic status (GDP *per capita*) across provinces in China, as shown in Figure 1. The results highlight a negative relationship between the proportion of dialect users and GDP *per capita* in the Beijing-Tianjin-Hebei and Yangtze River Delta regions, which might exacerbate socioeconomic disparities and MH risks for older adults. The negative correlation of dialects with the MH of older adults has persisted over time, which not only concerns the social development of older adults themselves but also affects the stability and development of their families and society (Kopinak, 2015; Li and Rose, 2017). Accordingly, further academic analysis is posed as a high priority, such as the multilingualism policy implemented by the Swiss government and the language resources protection project initiated by the Chinese Ministry of Education and State Language Commission (Kużelewska, 2016; Lixin, 2022).

The purpose of this study is to examine the relationship between dialect and MH among Chinese older adults. Despite some previous research on this topic (Wu et al., 2010; Zhong et al., 2017), the present study aims to promote the understanding of this topic by introducing income inequality (II) as a mediating variable and subjective well-being (SWB) as a moderating variable. The reasons we chose these two factors to participate in the potential mechanism between dialect and MH are as follows. On the one hand, the economic boom in China over the last 20 years has made II the largest social inequality and a distinguishing factor in determining individuals’ positions on the social ladder, there is also much relevant literature that considers II to be an important determinant in analyzing expected health disparities (Zimmerman and Bell, 2006; Ribeiro et al., 2017; Hao et al., 2021). On the other hand, SWB, which is academically conceptualized as an overall emotional and cognitive evaluation of an individual’s quality of life, is more relevant to MH than to socioeconomic status characteristics such as income, education, and occupation (Machado et al., 2019; Buecker et al., 2021). The present analysis takes these two factors into consideration and is consistent with the spirit of integrating economic and non-economic factors in the analysis of MH. Taken together, we aim to analyze the following: (1) the direct correlation between dialects and MH, (2) the mediation of II in the potential correlation between dialects and MH, and (3) the moderation between SWB in the above potential mechanism.

## 2. Literature review

In China, there are 7 major dialects and more than 100 sub-dialects (Kurpaska, 2010). MH is a psychological status that includes rational cognition, emotional stability, interpersonal harmony, and adaptation to changes in the growth process of an individual (Company-Córdoba et al., 2020). China has the largest population of older adults in the world, and the MH of older adults is often overlooked, despite improvements in living standards and healthcare services (Bao et al., 2022). MH disorders in older adults have led to reducing social and physical activities, nonconformity, self-grief, and decreasing quality of life (Wang et al., 2019). Identifying the determinants of MH is an academic necessity, and scholars have constructed a comprehensive analysis framework that takes individual, family, and social determinants into consideration, simultaneously (Kawachi and Berkman, 2001; Kramer et al., 2002). For instance, the analysis of individual attributes indicated that older males residing in rural areas, experiencing poverty, and possessing a lower level of education were at a higher risk of developing MH disorders compared to their affluent, female counterparts with higher educational attainment residing in urban areas (Wang et al., 2013; Case and Deaton, 2015).

The correlation between MH and traditional factors (e.g., income and gender) has been widely discussed (Conrey et al., 2005; Ravesteijn et al., 2016; Pot et al., 2018; Kose, 2020; Zaharchuk et al., 2021), while sporadic studies have focused solely on the direct association of MH with dialects (Shi et al., 2019; Ding et al., 2020; Miteva et al., 2022). On the one hand, several studies have revealed a direct positive correlation between these two factors, which supports the theory that dialects are an essential determinant in identifying relative position on the social ladder, and determines the quantity and quality of social resources for individuals (Hovey et al., 2006; Sentell et al., 2007). On the other hand, prior studies have concluded that dialects have a negative influence on MH (Ding and Hargraves, 2009; Zhao et al., 2022) and that dialect users have been systematically excluded from several occupations, which may contribute to their exclusion from society more broadly (Bhatia, 2018). Additionally, several experiments have also revealed clinical evidence of the correlation between dialect and MH. Some existing literature has linked the use of dialects to a higher likelihood of experiencing social exclusion and social pain (Tankosić and Dovchin, 2021). In turn, prolonged social pain has been linked with changes in the frontal cortex that might be associated with adverse MH outcomes (Berger and Sarnyai, 2015). Therefore, in contexts where dialect use leads to social exclusion, dialect use may also be linked with poor MH outcomes (Bhatia, 2018). Furthermore, the exact relationship between dialect use and MH outcomes remains unknown. We summarize two theoretical frameworks for analyzing the potential mechanism between the two selected factors: Planned Behavior Theory (PBT) and Norm Activation Theory (NAT; Fishbein and Ajzen, 1980). PBT posits that dialects promote a sense of identity, social connections, and emotional support (Schachter et al., 2012; Acemoglu et al., 2013). Thus, dialects may alleviate MH disorders by improving participation in the socialization process. This is more pronounced for older adults, as the impact of dialect on social integration has been found to rise with age (Jia, 2022). Empirical studies have revealed that limited proficiency in dialect represents one of the barriers to MH among older adults when they relocate to a new community characterized by a distinct dialect (Wong, 2013). Meanwhile, NAT suggests that dialects potentially influence MH in the following way: people internalize the use of dialects into their own behavioral norms and it inspires behavioral patterns that fit the identity of dialect speakers and influence MH (Baird and Sheffield, 2016; Soldevila-Domenech et al., 2021). However, both frameworks have limitations and fail to fully capture the comprehensive relationship between dialect use and MH.

Accordingly, there is a need to further study the potential mechanism between dialects and MH of older adults. In China, two factors deserve particular attention, one of which is II, which is defined as the difference between the income of the advantaged and the disadvantaged within a given group. Studies have shown that II causes significant socioeconomic disparities and increases the morbidity and mortality of MH disorders, especially regarding depression and anxiety (Matthew and Brodersen, 2018). II also acts as a mediator between other social determinants and MH (Grossman, 1972; Chiavegatto Filho et al., 2013; Burns, 2015), where the significant mediation influence could be captured in the potential mechanism, like gender, physical activity, and alcohol consumption (Lautenschlager et al., 2004; Kiely et al., 2019). Another factor is SWB, which has rarely been considered in previous studies (Shmotkin, 1998; Liu et al., 2022). Established empirical analysis has shown that strong SWB is positively associated with MH status in the older adult population (Magyar and Keyes, 2019), which is derived from the fact that SWB provides individuals with meaningful and coherent life experiences by accumulating positive life experiences (Tsaousis et al., 2007). SWB is more essential in the analysis of Chinese older adults because the established analysis shows that the proportion of older adults in China who are dissatisfied with their life status is significantly higher than in other countries, which might be an important determinant of the poor MH of older adults (Liang et al., 2020).

Taken together, two gaps in the literature can be identified: whether or not a correlation exists between dialect use and mental health and the nature of this correlation. Therefore, this study develops a moderated mediation model to investigate the potential relationship between dialects, II, SWB, and MH among Chinese older adults. Firstly, by including a mediation analysis, this study examines the potential mechanism that explains how dialects influence MH through the mediation of II. Secondly, the study includes a moderation analysis to examine the moderation of SWB in the mediating role of II on dialects and MH.

## 3. Hypotheses and conceptual model

### 3.1. Dialects and mental health

Chinese older adults are experiencing a growing prevalence of MH disorders, which presents them with various difficulties and obstacles. The determinants of MH are complex, encompassing economic, political, and cultural factors (Lund et al., 2018). In the literature review, we systematically summarize the established studies and identify two mechanisms between dialects and MH through two heterogeneous theoretical paths of Planned Behavior Theory (PBT) and Norm Activation Theory (NAT; Stöhr, 2015).

Dialects can directly impact the MH of older adults. Some scholars have pointed out that older adults with non-English speaking backgrounds living in the United States, including Chinese immigrants, exhibit relatively high levels of depression, potentially attributed to language differences (Lin et al., 2016). Previous studies have also identified significant levels of anxiety and depression among older adults who use dialects, leading to adverse impacts on their MH and overall quality of life (Seymour and Gale, 2005). Meanwhile, dialects, as a signal of different cultures and values and socioeconomic status, may play a deeper role in MH (Spector, 2004). For example, 42% of Vietnamese American families were found to experience “language isolation”, which has a significant impact on their level of acculturation and socioeconomic status. Consequently, these individuals face significant barriers to accessing MH care services (Kramer et al., 2002). In other words, dialects, as a symbol of a nation and culture, determine the relative position of older adults on the social ladder and influence the quality and quantity of their social resources and the accessibility of virtuous MH (Budiarsa, 2015). Therefore, the following hypothesis is proposed:

*Hypothesis 1 (H1). In a sociolinguistic context where a standard language dominates, dialects may be negatively associated with MH among older adults.*

### 3.2. The mediating effect of income inequality

Prior studies have also proven that dialects could indirectly impact the MH of older adults. Dialects may have a positive or negative influence on the socioeconomic status of older adults, resulting in economic disparities within society, systematically shaping II in a given society, and causing MH differences among older adults (Sturm and Gresenz, 2002). Several studies have proposed that language barriers may hinder the poor and ethnic minorities from sharing the necessary social capital, resulting in MH disparities (De Silva et al., 2007).

II has been identified as a crucial determinant of MH among older adults (Steele et al., 2007). The relationship between II and MH is complex because II may act as a mediator between dialects and MH among older adults. First, disadvantaged dialects are detrimental to MH because II exacerbates the negative effects of low educational attainment or social isolation on MH (Kim et al., 2013). Secondly, dialect and II lead to feelings of relative deprivation and lower self-esteem, which have been linked to MH disorders (Abe et al., 2012; Pickett and Wilkinson, 2015). Thirdly, dialect and II contribute to social exclusion and marginalization, leading to stress and decreased social support, which, in turn, negatively influence MH (Zhong et al., 2018; Brydsten et al., 2019).

The mediating effect of II on the relationship between dialects and MH highlights the importance of narrowing the II disparities among Chinese older adults and better understanding the need for dialect in socio-economic and cultural activities. Therefore, we propose the following hypothesis:

*Hypothesis 2 (H2). II mediates the correlation between dialects and MH.*

### 3.3. The moderating effect of subjective well-being

SWB is a crucial determinant of shaping positive psychology, encompassing an individual’s emotional reactions and overall judgments of life satisfaction (Headey et al., 1993). In previous studies, some psychologists have viewed SWB as a moderator of the potential mechanism between traditional factors and MH (Singhal and Rastogi, 2018). Although established analysis remains scarce, we propose the following explanatory pathway to analyze the moderation of SWB in the potential mechanism of dialect and MH (Tay et al., 2017).

Previous studies have suggested that language barriers are a crucial determinant in shaping MH disorders, especially for low self-esteem and anxiety (Solway et al., 2010; Ohtani et al., 2015). Based on this point, we propose that SWB might moderate the relationship between dialects and MH, as higher SWB could reduce communication difficulties and anxiety, reduce the language barriers, and shape good MH for older adults (Diener and Lucas, 2000; Gargiulo and Stokes, 2009). Sociologists have also posited that older adults with high SWB have increased confidence and are better equipped to improve their social competence, promoting a harmonious social environment and systematically improving MH (Tugade and Fredrickson, 2004).

Furthermore, the moderation of SWB for the above potential mechanism may vary in different situations. Prior studies have suggested that the effects of dialects on II vary based on SWB levels as there is a positive and significant relationship between dialects and II under high SWB, but a non-significant relationship under low SWB (Danzer and Danzer, 2016). Furthermore, research has identified a significant negative impact of II on SWB, particularly among the older adult population (Headey and Wooden, 2004). Accordingly, we propose the following hypothesis:

*Hypothesis 3 (H3). SWB moderates the negative impact of dialects on MH.*

*Hypothesis 4 (H4). SWB moderates the mediating effect of II on the relationship between dialect and MH.*

**Figure 2:**
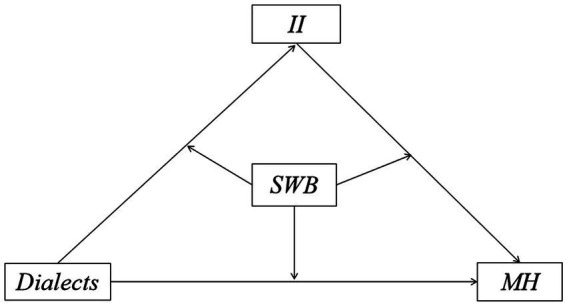
Theoretical Framework of the Study.

The whole potential mechanism takes dialects and MH as the independent and dependent variables and the II and SWB as the mediator and moderator, as shown in Figure 2.

## 4. Methodology

### 4.1. Data

This study utilizes data from the China Family Panel Studies (CFPS), an ongoing periodic national survey covering substantial numbers of respondents from all 31 provinces of China. The CFPS was designed by the China Social Science Survey Center at Peking University and was widely used in multidisciplinary analysis (Xu et al., 2014; Maireche, 2015). We used the latest CFPS 2020 survey to ensure the reliability of the study findings. To ensure consistency in the sample, only respondents aged 60 years or older were retained, resulting in a final valid sample of 4,420 after the exclusion of missing values. Written informed consent was obtained from all participants.

### 4.2. Measures

#### 4.2.1. Independent variable

The independent variable was dialects. Respondents were asked “Which of the following languages is mainly used?,” and the available answers included “Dialect” and “Mandarin.” Of these, the number of older adults using “Dialect” was 1755, and the number of older adults using “Mandarin” was 2665. The Dialect group was recorded as 1 and the Mandarin group was recorded as 0, and the mean value of the full sample was 0.397.

#### 4.2.2. Dependent variable

Mental health was the dependent variable, and it was measured using the rigorous Center for Epidemiological Studies-Depression (CES-D) scale developed by Radloff (1977). In consideration of the psychological characteristics of Chinese older adults, CFPS retained the eight items in CES-D, which are as follows: in the past week, have you felt (1) upset, (2) I had trouble keeping my mind on what I was doing, (3) my sleep was restless, (4) happy, (5) lonely, (6) satisfied, (7) sad, (8) desperate. The response format was a 4-point scale (1 = always, 2 = often, 3 = sometimes, and 4 = never). To ensure the consistency of the scoring direction, we reordered the fourth and the sixth items so that all the items followed the rule that the higher the score, the worse the MH. The mean score of CES-D was 26.509.

#### 4.2.3. Mediating variable

Income inequality was the mediating variable, which participated in the potential correlation between dialects and MH. II was conceptualized as inequality in income between populations, which was expressed by averaging the sum of two factors, individual-level II and group-level II. Individual-level II is averaged using the following equation:


$$\{\begin{array}{c} II_{i}=\overset{n}{\underset{i=1}{\sum }}\left(\frac{I_{i,m}-\bar{I_{m}}}{i}\right)\left(a\right)\\ II_{g}=\overset{o}{\underset{k=1}{\sum }}X_{k}Y_{k}+2\overset{o}{\underset{k=1}{\sum }}X_{k}\left(1-\upsilon_{i}\right)-1\;\;\left(b\right)\\ II=\left(II_{i}+II_{g}\right)/2\;\;\;\left(c\right) \end{array}$$

Where, in equation (a), I_i,m_ denotes the income of individual i in region m and $\bar{\;I_{m}}$ is the weighted mean value of income in region m. In equation (b), x represents the population proportion of each group, y indicates the income proportion of each group, and υ denotes the cumulative income proportion of each group, all of which were divided into 10 groups at the 10% quantile. Equation (a) denotes the final II, which takes the value of II_i_ and II_g_ into consideration, simultaneously. We defined II as above with reference to several economists (Roser and Ortiz-Ospina, 2013; Dabla-Norris et al., 2015).

#### 4.2.4. Moderating variable and control variables

Subjective well-being was the moderating variable, which participated in the potential correlation between dialects and MH. SWB was expressed according to the respondent’s perception of their life in response to the question “How satisfied are you with your current state of life?.” SWB was a continuous variable with values ranging from 0 to 10, where a larger value indicated that respondents were more satisfied with their life status, with a mean value of 7.779 in the full sample.

Additionally, to eliminate the potential estimated bias, the age (age of Respondents), gender (0 = female，1 = male), years of education (years of education of respondents), marriage (current marital status), household size (number of family members), family economic status (assessed by considering various factors such as daily living expenses, accumulation of fixed assets, and current household income, and then assigned weights and combined to calculate a comprehensive evaluation score), social security (ratings of social security systems by members of society), and pension insurance (the proportion of older adults who receive pension insurance) were taken as the control variables. Basic variables descriptions are presented in Table 1.

**Table 1:** Basic variables description^a^.

Variable	Operationalization	Source
Gender	0 = female，1 = male	CFPS^b^
Age	Age of Respondents (60–95)	CFPS
Years of education	Years of education of respondents (0–16)	CFPS
Marriage	Current marital status (1 = unmarried，2 = married，3 = cohabiting，4 = divorced，5 = widowed)	CFPS
Household size	Number of family members (1–15)	CFPS
Family economic status	Assessed by considering various factors such as daily living expenses, accumulation of fixed assets, and current household income, and then assigned weights and combined to calculate a comprehensive evaluation score (1–5)	CFPS
Social security	Ratings of social security systems by members of society (1–10)	CFPS
Pension insurance	The proportion of older adults who receive pension insurance (0–1)	CFPS
Dialects	Which of the following languages is mainly used (0 = Mandarin, 1 = Dialect)	CFPS
II	The sum of individual-level and group-level income inequality (0–37)	CFPS
SWB	How satisfied are you with your current state of life (0–10)	CFPS
MH	Respondents’ mental health, calculated by CES-D 8 scale^c^ (8–32)	CFPS

a *N* = 4420.

b CFPS is available at www.isss.pku.edu.cn/cfps/.

c Center for Epidemiological Studies Depression (CES-D 8) is a commonly used scale to measure mental health.

### 4.3. Analysis

We used MPLUS 8.2 to analyze the potential relationship between dialect and MH in Chinese older adults. To achieve this, we developed a structural equation model (SEM) based on a mediator of II and a moderator of SWB (Hayes, 2012).

Firstly, descriptive statistics were used to initially capture the relationship of the selected variables and reveal potential relationships between dialect, II, SWB, and MH. Based on the literature, we hypothesized that II and SWB indirectly mediate and moderate the relationship between dialect and MH, simultaneously. To test this hypothesis, we verified the relationship between these four factors by statistical test of correlation, where a *t*-test was used for continuous variables and a χ^2^ test was used for categorical variables.

**Figure 3:**
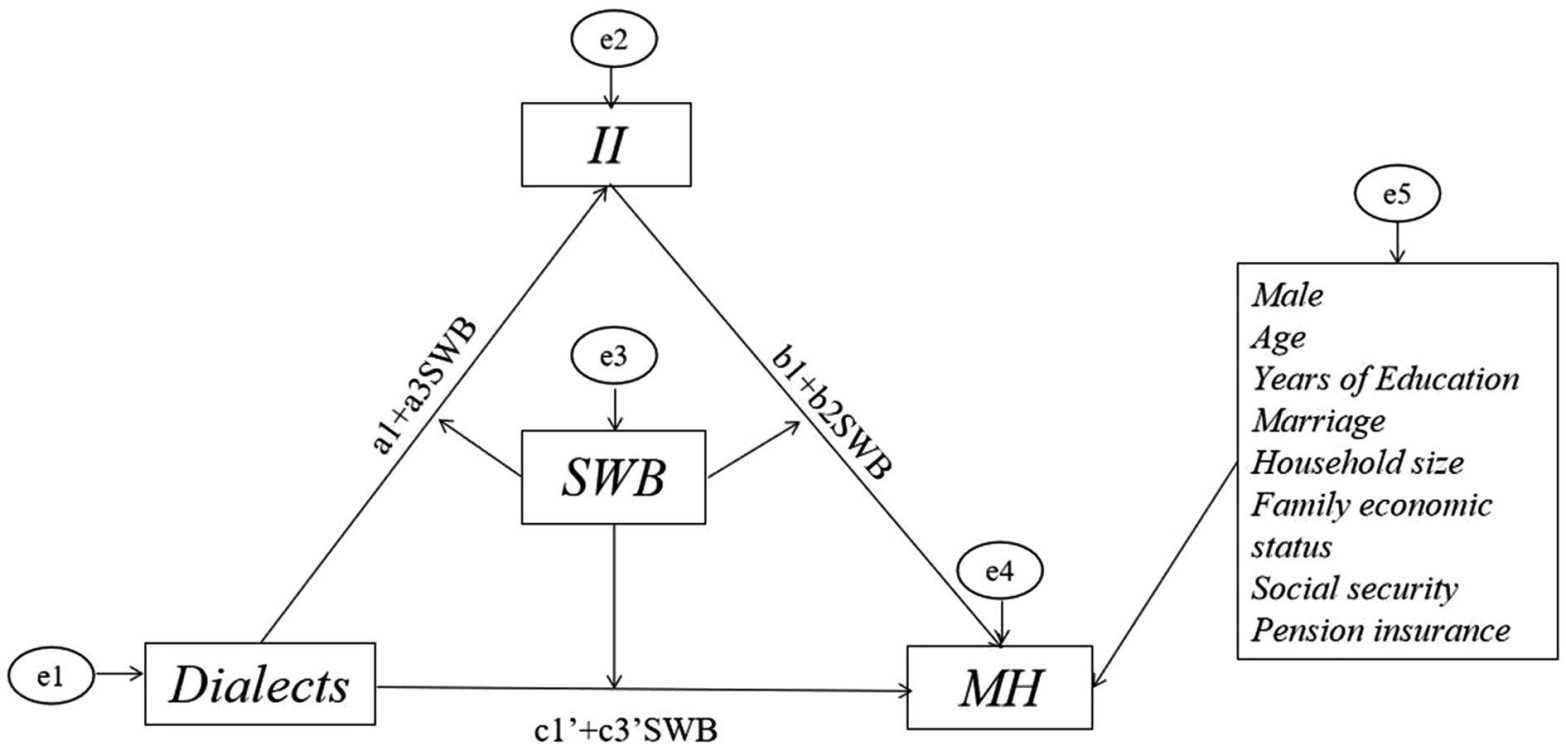
Moderated mediating effects based on structural equation modeling^ab^. ^a^The moderating variable SWB was adjusted for the three paths: dialects→II, dialects→MH, II → MH. ^b^SWB served as the moderating variable in the association between dialects and MH, with a portion of the moderating effect being mediated by the mediating variable II. The indirect moderating effect could be observed through the coefficient pertaining to SWB in the equation (a1 + a3SWB)(b1 + b2SWB), while the direct moderating effect was represented by the coefficient of c1’ + c3’SWB.

Secondly, we designed a SEM to describe the potential relationship between dialect and MH and sequentially demonstrated a moderated mediation effect in Figure 3, where the mediating variable was II and the moderating variable was SWB. We used the specific code of modindices in MPLUS to optimize our model through the modification index (MI). We also estimated the direct and indirect effects of the correlation between dialect and MH. As for the multivariate non-normality, bootstraps (5000 times) was used as the repeated sampling technology in our research to reduce bias. Meanwhile, we took all covariates into consideration.

## 5. Results

### 5.1. Common method bias test

The present study involves the use of self-reported data to obtain several relative variables (II, SWB, MH), which could lead to biased estimates. To address the above issues, a common method bias test was conducted. Harman’s single factor test showed that there were three factors with Eigen roots greater than one, and the first principal component accounted for 31.47% of the total unexplained residuals. This finding suggests that the common method bias did not account for a significant proportion of the variance in the measured constructs, as the first principal component explained less than the recommended threshold of 40% (Podsakoff et al., 2003).

In summary, it can be concluded that the potential bias resulting from the use of self-reported data in the selected sample did not significantly affect the empirical estimation. Therefore, the results of this study can be considered reliable and valid.

### 5.2. Descriptive statistics and correlation analysis

In order to preliminarily verify the validity of the SEM, we conducted data description and correlation analysis, as shown in Table 2.

**Table 2:** Mental health outcomes, income inequality, subjective well-being, and other control variables among different groups of Chinese older adults^a^.

Variables	Full (4420)		Mandarin (2665)		Dialect (1755)		*t*-test^b^
	Mean	SD	Mean	SD	Mean	SD	
Male	0.523	0.500	0.526	0.499	0.520	0.500	0.694
Age	68.035	5.749	67.609	5.642	68.683	5.850	0.000***
Years of education	5.615	4.992	5.826	5.760	3.731	5.456	0.000***
Marriage	2.373	0.978	2.427	1.044	2.338	0.930	0.003***
Household size	3.776	2.156	4.116	1.945	3.552	2.402	0.000***
Family economic status	3.202	1.148	3.230	1.188	3.183	1.120	0.188
Social security	5.032	3.107	5.149	3.121	4.855	3.092	0.002***
Pension insurance	0.682	0.466	0.742	0.438	0.642	0.479	0.000***
II	0.550	1.748	0.594	1.645	0.482	1.812	0.037**
SWB	7.779	2.140	7.898	2.294	7.597	2.025	0.000***
MH	26.509	4.481	26.720	4.507	26.190	4.452	0.000***

a *p < 0.05, **p < 0.01, ***p < 0.001.

b The χ^2^ test and *t*-test were used for categorical and continuous variables, respectively.

The results of the descriptive statistics for the full sample and for the two subgroups (Dialect group and Mandarin group) are shown simultaneously. Nearly all the selected variables are systematically higher in the Mandarin group compared to the Dialect group. The three selected variables are worth particular attention, where II, SWB, and MH all share a higher mean value in the Mandarin group compared to the Dialect group, which is statistically significant.

Table 3 presents the results of the correlation analysis, which indicate that the relationships between the main variables are generally statistically significant, except for the correlation between II and SWB. More specifically, dialects were found to be significantly and negatively associated with II (r = −0.031, *p* < 0.05), SWB (r = −0.069, *p* < 0.01), and MH (r = −0.058, *p* < 0.01), respectively. This suggests that dialect users are systematically associated with greater II, lower SWB, and lower MH. Moreover, there is no direct evidence of a statistically significant relationship between SWB and II. Additionally, MH was found to be negatively related to II (r = −0.066, *p* < 0.01) but positively related to SWB (r = 0.324, *p* < 0.01). Taken together, the above findings prove the necessity of constructing SEM to analyze the potential mechanism between dialects and MH.

**Table 3:** Pearson correlation analysis for all selected variables^a^^,^^b^.

Variables	1.	2.	3.	4.	5.	6.	7.	8.	9.	10.	11.	12.
1.Male	—											
2.Age	0.004	—										
3.Years of Education	0.239 **	−0.107 **	—									
4.Marriage	−0.165 **	0.228 **	−0.097 **	—								
5.Household size	0.029	−0.137 **	−0.075	−0.084 **	—							
6.Family economic status	0.007	0.108 **	−0.034 *	−0.014	−0.021	—						
7.Social security	−0.033 *	−0.075 **	0.040 **	0.002	0.024	−0.040 **	—					
8.Pension insurance	0.003	0.066 *	−0.090 **	0.024	0.026	0.027	−0.006	—				
9.Dialects	−0.006	0.091 **	−0.179 **	0.045 **	0.128 **	0.020	−0.046 **	0.105 **	—			
10.II	−0.015	0.048 **	0.017	0.009	−0.013	−0.002	0.003	−0.011	−0.031 *	—		
11.SWB	0.011	0.070 **	0.048 **	−0.050 **	−0.025	0.243 **	−0.028	0.024	−0.069 **	−0.005	—	
12.MH	0.154 **	0.002	0.160 **	−0.104 **	−0.001	0.146 **	−0.085 **	−0.002	−0.058 **	−0.066 **	0.324 **	—

a *p < 0.05, **p < 0.01.

b The χ^2^ test and *t*-test were used for categorical and continuous variables, repectively.

### 5.3. The mediating effects of income inequality

**Figure 4:**
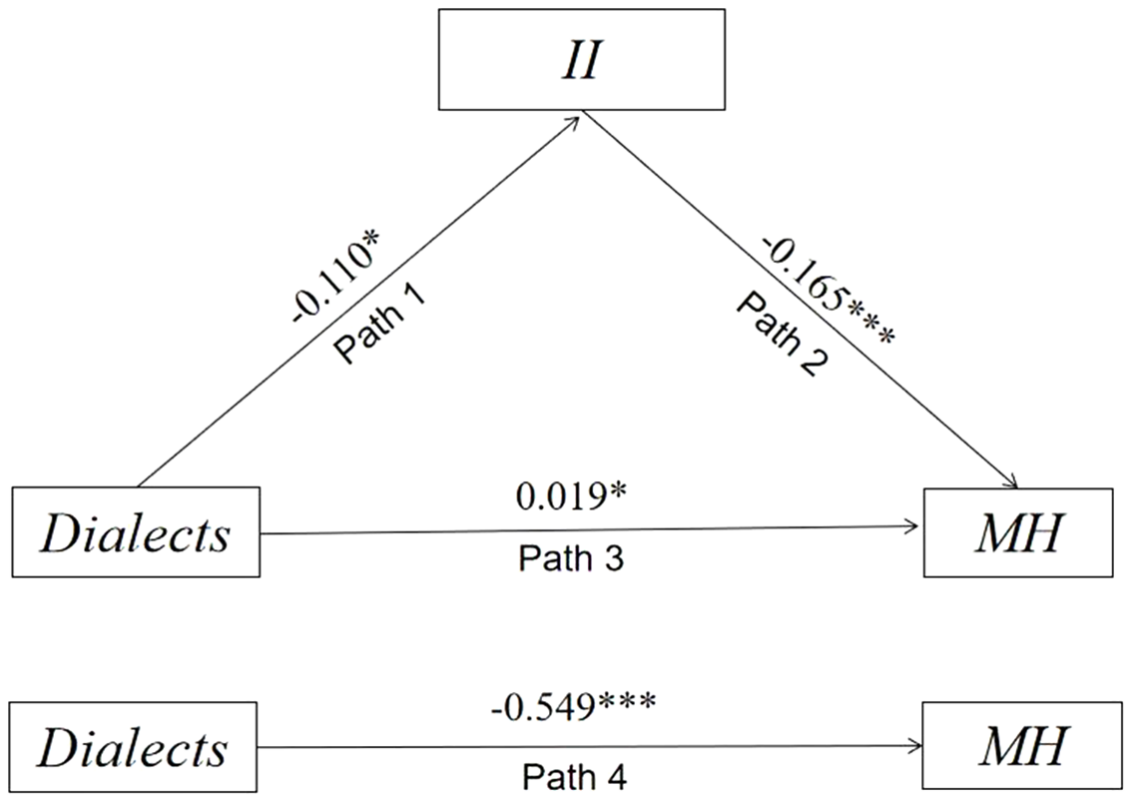
Estimation of mediation model^a^. ^a^Total effects = Path 3 + Path 4 = −0.530, statistically significant.

To investigate whether II mediates the effect of dialects on MH, we employed MPLUS 8.2. The results of the analysis are presented in Table 4 and Figure 4.

**Table 4:** Mediating effect and 95% confidence interval.

Path		Effect Estimation	95%CI	
Path 1	Dialects→II	−0.110	−0.217	−0.017
Path 2	II → MH	−0.165	−0.241	−0.100
Path 3^a^	Dialects → II → MH	0.019	0.010	0.045
Path 4^b^	Dialects→MH	−0.549	−0.819	−0.285
Total Effects		−0.530	0.000	0.045

a Model 2 represents the mediating effect of II on the relationship between dialects and MH.

b Model 1 represents the direct effect of dialect on MH.

On the one hand, the direct correlation between dialects and MH was found to be statistically significant and negative (coefficient = −0.549, 95% CI = [−0.819, −0.285]), which is consistent with the data description and supports several established analyses (Chen et al., 2005). On the other hand, the correlation between dialects and MH was found to be partially mediated by II; more specifically, II positively mediates the potential correlation between dialects and MH (coefficient = 0.019, 95% CI = [0.010, 0.045]). Thus, H1 is supported.

In summary, our findings provide evidence that II plays a significant mediating role in the relationship between dialects and MH, as demonstrated by the significant total indirect effect. On the one hand, they reveal the systematic relationship between dialect use and MH disorders, and on the other hand, they support that the MH gap due to dialect use is caused by the disparity in socioeconomic elements (Zhang and Zhang, 2017).

### 5.4. The moderating effects of subjective well-being

We set a moderated mediation model to capture the potential moderating effects of SWB on the mediated model. The results are presented in Table 5.

**Table 5:** Results of the moderating effect testing.

	Model 3				Model 4			
	β	SD	95%CI	*t*	β	SD	95%CI	*t*
Dialects	−0.113	0.054	[−0.222, −0.011]	−2.156*	−0.354	0,130	[−0.608, −0.097]	−2.713**
SWB	0.016	0.017	[0.008, 0.031]	1.110*	0.721	0.040	[0.652, 0.710]	18.073***
II					−0.171	0.036	[−0.243, −0.201]	−4.726***
Dialects x SWB	−0.050	0.025	[−0.131, −0.004]	−2.128*	−0.126	0.060	[−0.284, −0.010]	−2.132*
R	0.045				0.334			
R^2^	0.012				0.112			
F	3.042*				136.791***			

Firstly, we tested whether the mediation model remained robust after including SWB. As shown in the table, the direct effect of dialects on MH in Model 4 remained statistically significant (coefficient = −0.354, 95% CI = [−0.608, −0.097]) and the indirect effect of dialects on MH through II remained statistically significant (coefficient = 0.016, 95% CI = [0.008, 0.031]).

**Figure 5:**
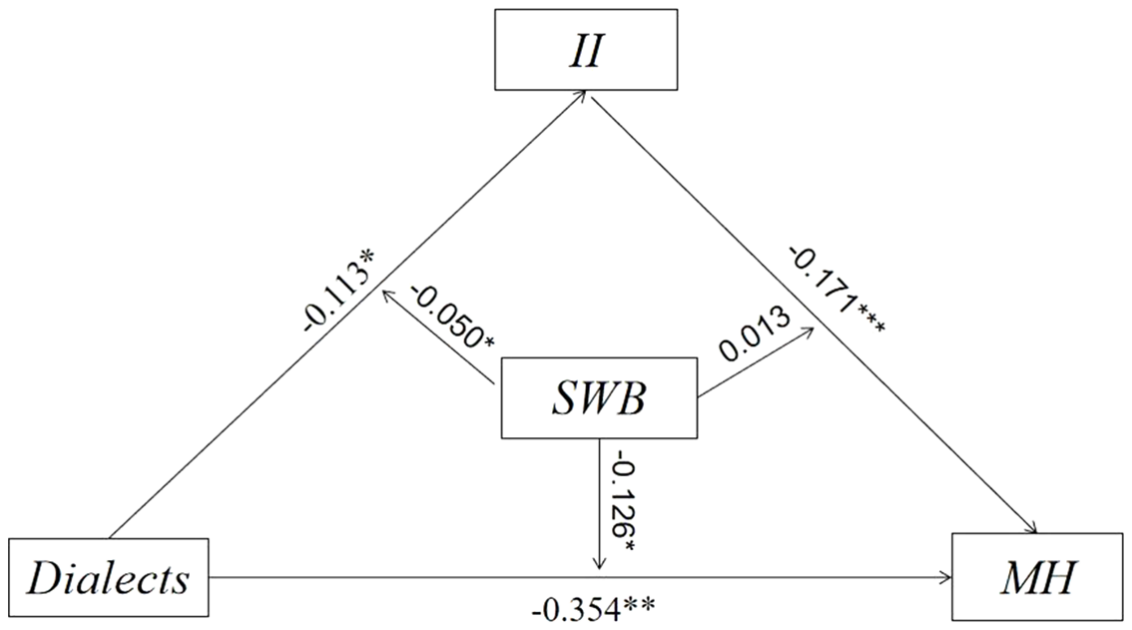
Estimation of modified model^a^. ^a^The moderating effect on the upper left is significant, the moderating effect on the upper right is not significant, and the total moderating effect of dialects on MH through II is significant.

Secondly, we examined whether SWB moderated the mediation model in Figure 5. The results indicate that the moderating effects of SWB on the direct effect are statistically significant and negative (coefficient = −0.126, 95% CI = [−0.284, −0.010]), suggesting that the adverse effects of dialect on MH are more severe for those with lower SWB. Additionally, the moderating effects of SWB on the total indirect effect of dialects on MH through II are statistically significant and negative (coefficient = −0.050, 95% CI = [−0.131, −0.004]), indicating that the indirect effect is stronger for individuals with higher levels of SWB. The moderating effect of SWB on the indirect effects of II and MH is not significant. Overall, the moderating effect of SWB in the mediating effect of II on dialect and MH is significant. These findings provide support for H3 and H4, suggesting that SWB moderates not only the direct relationship between dialect and MH but also the indirect effect of II on dialect and MH.

**Figure 6:**
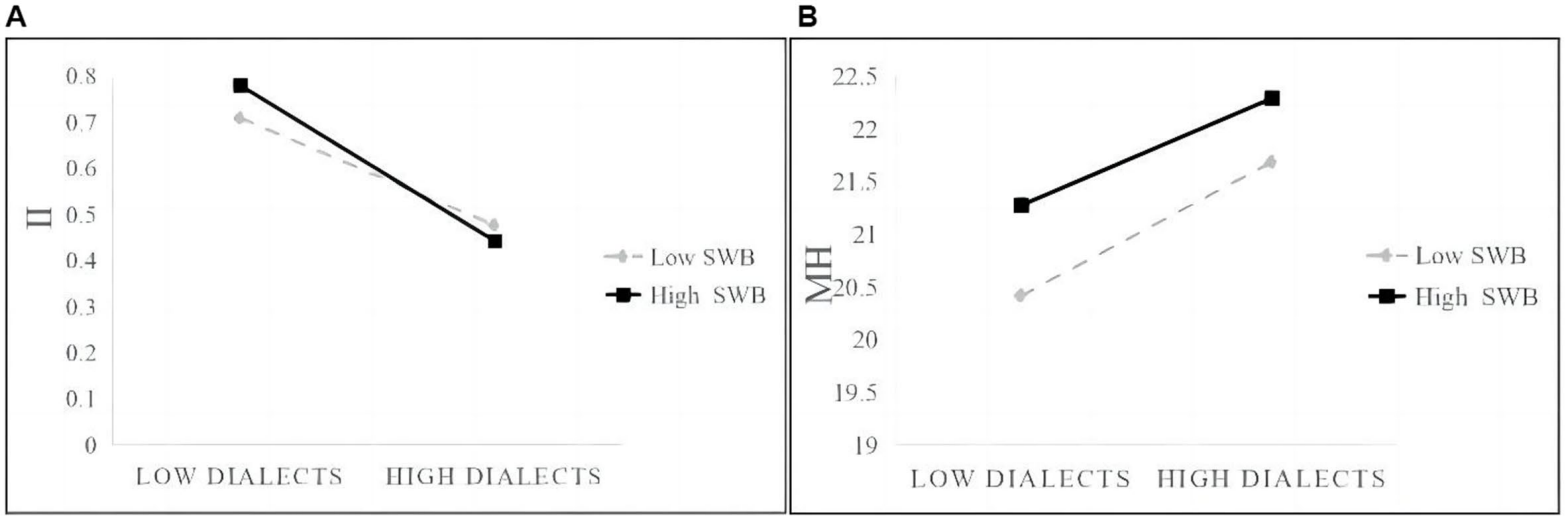
**(A)** SWB moderated effects on indirect effects; **(B)** SWB moderated effects on direct effects.

To further illustrate the moderating effects of SWB, we show the differences in moderation at different MH, II levels (mean minus standard deviation, mean, mean plus standard deviation) in Table 6 and Figure 6 (Aiken et al., 1991). We first divided older adult participants into three groups based on their SWB levels: low, middle, and high. Our findings show that the negative effect of dialects on MH is more significant in the middle (Effect = −0.354, 95% CI = [−0.638, −0.097]) and high (Effect = −0.624, 95% CI = [−0.985, −0.262]) groups compared to the low group (Effect = −0.081, 95% CI = [−0.435, 0.274]). Moreover, the moderation influence of SWB on the mediated model was found to be more significant in the middle (Effect = 0.020, 95% CI = [0.002, 0.046]) and high groups (Effect = 0.039, 95% CI = [0.015, 0.071]) compared to the low group (Effect = 0.008, 95% CI = [−0.022,0.132]).

**Table 6:** Results of the effect of dialect on MH at different levels of SWB.

Moderator	Direct effect of dialects			Indirect effect of II		
	Effect	BootSE	95%CI	Effect	BootSE	95%CI
eff1(M-1SD)	−0.081	0.181	[−0.435, 0.274]	0.008	0.014	[−0.022, 0.132]
eff2(M)	−0.354	0.130	[−0.638, −0.097]	0.020	0.011	[0.002, 0.046]
eff3(M + 1SD)	−0.624	0.185	[−0.985, −0.262]	0.039	0.014	[0.015, 0.071]

Taken together, the conclusion indicates that SWB significantly and negatively moderates the direct and indirect effects, simultaneously.

### 5.5. Robustness tests

**Figure 7:**
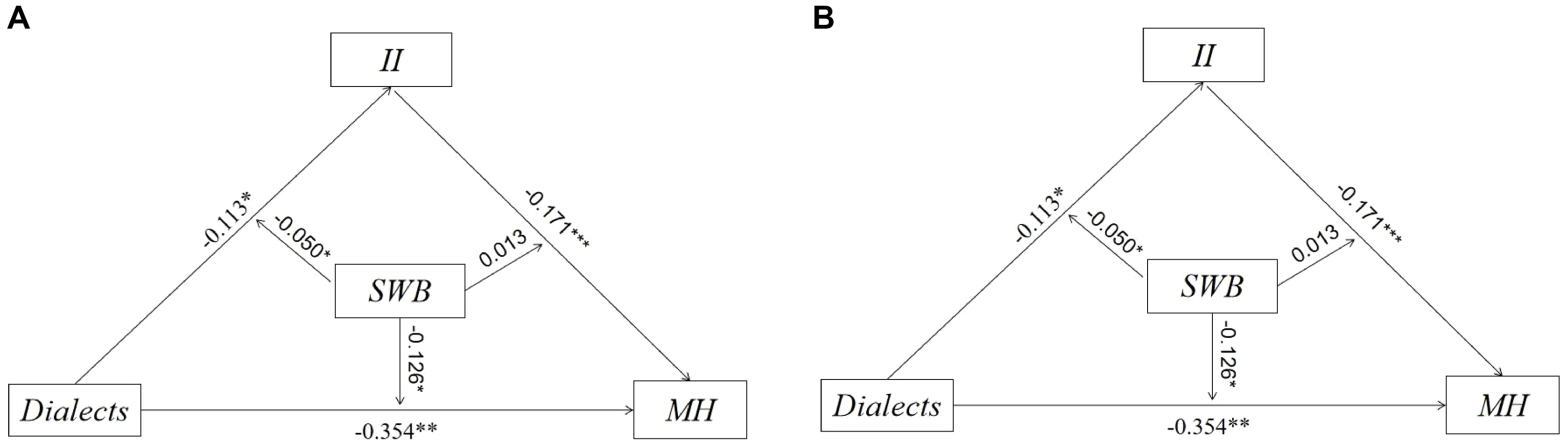
**(A)** Robustness tests (Bootstrap = 1000); **(B)** Robustness tests (Bootstrap = 5000).

To ensure the reliability of the findings, we employed the Bootstrap method to test the moderated mediation model with different sample sizes (1000 times and 5000 times). The confidence intervals of the upper and lower limits were examined to determine whether the model was valid. The results of Figure 7 show that the coefficients obtained from Bootstrap 1000 and Bootstrap 5000 are consistent. Taken together, the findings are robust.

### 5.6. Heterogeneity analysis

**Figure 8:**
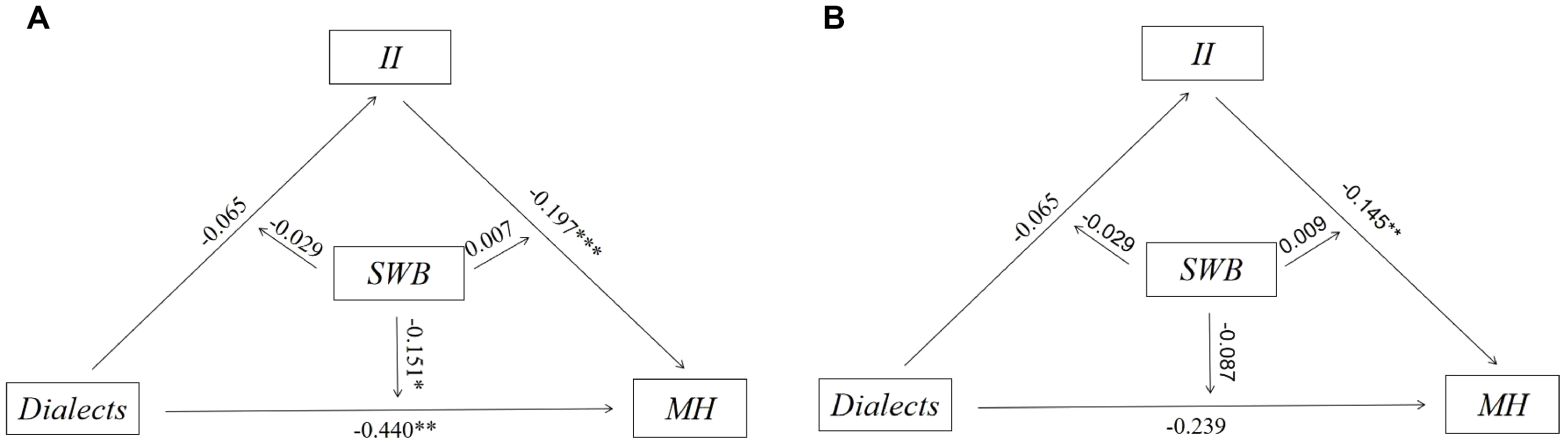
**(A)** Heterogeneity of effect of dialects in the younger participants on MH^a^; **(B)** Heterogeneity of effect of dialects in the older participants on MH^b^. ^a^The younger participants were defined as those who were younger than the mean age of 68 years. ^b^The older participants were defined as those who were older than the mean age of 68 years.

**Figure 9:**
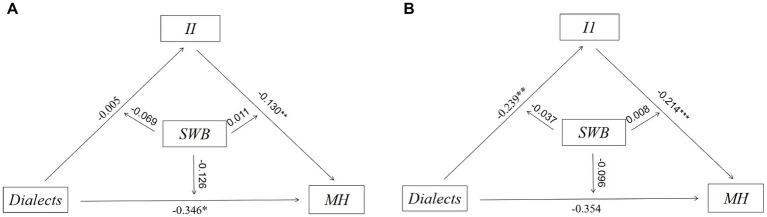
**(A)** Heterogeneity of effect of dialects in the male group on MH; **(B)** Heterogeneity of effect of dialects in the female group on MH.

**Figure 10:**
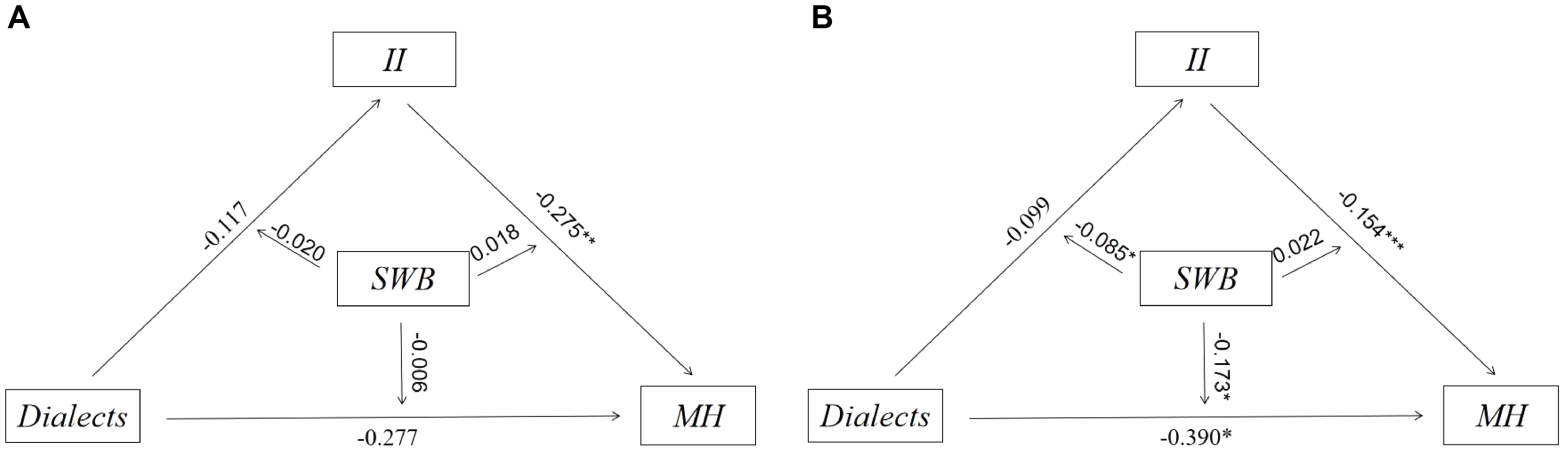
**(A)** Heterogeneity of effect of dialects in the low education group on MH^a^; **(B)** Heterogeneity of effect of dialects in the high education group on MH^b^. ^a^The low education group was defined as those with less than the mean of 5 years of education. ^b^The high education group was defined as those with more than the mean of 5 years of education.

To examine the heterogeneous influence across different demographic groups, we conducted a heterogeneity analysis. The data pertaining to age and years of education were partitioned into two distinct groups based on the application of the mean as a differentiating parameter; the results are presented in Figures 8–10. First, dialects were found to significantly predict lower MH in younger participants (aged 60–68; coefficient = −0.440, *p* < 0.01), while the effect was identified as not significant in the older participants (aged 69–95). Second, the negative correlation between dialects and MH was found to be more significant in the male group (coefficient = −0.346, *p* < 0.05) compared to the female group. Third, the high education group was found to be more susceptible to the negative effects of dialects (coefficient = −0.390, *p* < 0.05) compared to the low education group.

These findings are in line with previous research, showing that the younger participants group, the male group, and the high education group tended to have more negative outcomes for MH (Schnittker, 2005; Tribe and Lane, 2009; Cornaglia et al., 2015). However, the results of this study add to the literature by demonstrating that the use of dialects may exacerbate the disparities in MH outcomes. Specifically, dialects were found to have a significant negative correlation with MH in the younger participants, male, and high education groups, suggesting that individuals in these groups are more vulnerable to the negative effects of dialects.

## 6. Discussion

Two academic gaps were identified in the existing studies: the direct correlation and the potential mechanism between dialects and MH (Conner and Heywood-Everett, 1998; Stöhr, 2015). To narrow these academic gaps, this study investigates the direct correlation and potential mechanism between dialects and the MH of Chinese older adults, considering II and SWB as mediator and moderator, respectively, and we have captured the following meaningful conclusions.

### 6.1. The direct effect of dialects on the mental health of older adults

Dialects have been recognized as an important socioeconomic factor that may cause psychological inequality for older adults (Keyes, 2005). The direct effect of dialects on the MH of older adults was explored, and the results presented in Table 5 show that in the Chinese context, where there is a clear standard language spoken by the majority of the population and employed exclusively in public life, dialect use was found to have a significant negative influence on MH (coefficient = −0.549, 95% CI = [−0.819, −0.285]). These findings differ from some previous studies conducted in Western countries, such as the United States and the United Kingdom, which found that dialects positively influence MH (Sentell et al., 2007; Gong et al., 2011). This suggests that there are two opposite paths of dialect influence on MH, namely, the shaping of social capital and social barriers.

The shaping of social capital occurs when dialect speakers form advantaged social groups, similar to Dürkheim’s concept of organic solidarity, which improves individual socioeconomic status and positively affects MH (Durkheim and Schmidts, 2017). On the other hand, the shaping of social barriers occurs when dialect speakers are excluded from mainstream social culture, which is similar to the concept of social exclusion by René Lenoir (Rawal, 2008), resulting in MH disorders. Kachin people who use English and are socially integrated into American culture were found to be psychologically healthier than Mexican-Americans who reject American culture (Ortiz and Arce, 1984). Proficiency in the local dialect among Norwegian immigrants was found to facilitate their social integration, resulting in improved MH outcomes (Dalgard and Thapa, 2007). The studies conducted so far in America and Europe belong to the former, while the current study from China aligns more with the latter.

Therefore, this study highlights the need for Chinese policymakers to pay special attention to dialect-using populations, given the negative effect of dialects on the MH of older adults. Following the policy design, they could reduce the psychological inequality caused by dialects and improve the MH of older adults.

### 6.2. The mediating effect of income inequality

The empirical results reveal that II partially mediates the relationship between dialects and the MH of older adults. Specifically, the total indirect effects were found to be statistically significant, both in the mediated model (coefficient = 0.019, 95%CI = [0.010, 0.045]) and in the moderated mediation (coefficient = 0.016, 95%CI = [0.008, 0.031]). These results suggest that regulating II could be an effective strategy for alleviating the negative effects of dialects on MH.

Given the enormous burden of MH disorders in older adults in China (Bird et al., 2001; Crawford et al., 2013), it is essential to develop effective interventions to mitigate the negative effects of dialects on their MH. Although previous empirical studies have identified various measures, such as promoting primary care, improving mental health treatment rates, and enriching psychologist resource allocation, it remains problematic from a socioeconomic point of view, especially for II (Henderson and Louhiala-Salminen, 2011; Cuesta and Budría, 2015).

As a government-led country, the Chinese government needs to develop national policies that promote scientific and reasonable wealth distribution and further reduce the socioeconomic status disparity in Chinese older adults. Additionally, adjusting the II among older adult residents could also be an effective strategy for mitigating the negative impact of dialects on MH. It is a priority for the Chinese government to narrow the II gap among the older adult population, particularly through pension allocation, fair insurance systems, and quality pension services (Wang et al., 2014).

### 6.3. The moderating effect of subjective well-being

We explored the potential moderating effects of SWB on the relationship between dialects, II, and the MH of older adults in China. Our results show that SWB functions as a moderator to reduce the negative impact of dialect on MH, with a statistically significant moderating effect of −0.126 (95% CI = [−0.284, −0.010]). Additionally, the moderating effects of SWB on the mediating effect of II on dialect and MH were found to be −0.050, making them statistically significant (95% CI = [−0.131, −0.004]).

The results verify that SWB functions as a moderator to reduce the negative impact of dialect on MH. The negative moderation of SWB on MH disorders caused by dialects is a double-edged sword. On the one hand, it implies that the MH disparity among different social classes could be magnified and could shape new health inequalities. On the other hand, this positive moderation provides a solution strategy for the Chinese government to alleviate MH disorders in older adults. Our findings suggest that offering older adults improved SWB could shape positive interpersonal relationships and harmonious social atmospheres, which promotes the elimination of language barriers and the protection of MH health (von Humboldt et al., 2015; Lee and Cagle, 2018).

These results present significant implications for policymakers and healthcare professionals in China as they highlight the importance of implementing policies and interventions aimed at improving the SWB of older adults, particularly those living in areas with high dialect usage. We suggest that the Chinese government should aim to improve the SWB of older adults to alleviate MH disorders by optimizing living conditions, encouraging interpersonal interaction, and creating a positive social atmosphere (Zhu et al., 2021).

### 6.4. Limitations and further research

The study aims to narrow the research gap and advance scientific understanding of the comprehensive correlation between dialects and MH; however, several limitations must be explained carefully.

(1) Data limitations. Cross-sectional data may be affected by causal estimation bias. The current study focuses on correlation estimation because it does not control for systematic statistical bias caused by errors such as reverse causality, omitted variables, and sample self-selection. Future research could employ cohort data and scientifically design causal estimation models to provide a more robust test of causality.
(2) Variable limitations. Several related variables were from self-report questionnaires, which may induce unintentional reporting bias. We recommend that future studies use more rigorous clinical methods to measure individual psychological states.

Despite these limitations, the present study provides a framework for the potential correlation between dialects and MH, and accordingly, it shares substantial important implications for both theory and practice.

## 7. Conclusion

The present study captures the relationship between dialects and MH, and several meaningful conclusions are drawn: (1) dialects have a significant negative effect on the MH of older adults, (2) II mediates the relationship between dialects and the MH of older adults, and (3) SWB moderates the effects of dialects on MH and the effect of II on dialect and MH.

## Data availability statement

The original contributions presented in the study are included in the article/supplementary material, further inquiries can be directed to the corresponding author.

## Ethics statement

The studies involving human participants were reviewed and approved by the Ethics Committee of the School of Shaanxi Normal University Shaanxi Normal University. The patients/participants provided their written informed consent to participate in this study.

## Author contributions

TL and JL contributed to the conception and design of the study. TL and XK organized the database and revised the manuscript format. TL, JL, and XK performed the statistical analysis. TL wrote the first draft of the manuscript. All authors contributed to the article and approved the submitted version.

## Funding

This research was funded by the National Social Science Fund of China (18BYY049) and Fundamental Research Funds for the Central Universities (2021TS084).

## Conflict of interest

The authors declare that the research was conducted in the absence of any commercial or financial relationships that could be construed as a potential conflict of interest.

## Publisher’s note

All claims expressed in this article are solely those of the authors and do not necessarily represent those of their affiliated organizations, or those of the publisher, the editors and the reviewers. Any product that may be evaluated in this article, or claim that may be made by its manufacturer, is not guaranteed or endorsed by the publisher.
